# Health Work in the States

**Published:** 1924-10

**Authors:** 


### HEALTH WORK IN THE STATES.

### A BRITISH MEDICAL OFFICER'S NOTES.

We referred, in an article last month, to the "interchanges of health officials" established by the League of Nations and made possible by the International Health Board of the Rockefeller Foundation shouldering the expense. Dr. Charles Porter, the Medical Officer of Health for Marylebone, who visited the States in company with other medical officers, has furnished us with some of his notes of the visit, which show that he was an interested and interesting observer. In speaking of the officers in charge of the main divisions of the Federal Public Health Service, he says that these officers are, like all the members of the service, most carefully selected and, having had, most of them, opportunities of travelling widely in connection with their duties not only throughout the States, but to other parts of the world, are in general men of broad outlook.

### The Health Organisations.

This service, says Dr. Porter, is quite different from any organisation at home, and is, so far as he is aware, unique in the world :—

In our minds the idea was, not unnaturally, that the United States Public Health Service was the central government service, and that, to a very large extent, the States health services were subject to it, took directions from it, and were under its supervision. This it was shown was not so, the United States Service being more or less definitely a separate service created primarily for the purpose of protecting the United States, as a land, from invasion by disease, and, secondly, for guarding each State from disease and health threats from the others. With public health work within the States the Federal Service has little if any power to interfere. Each State has, of course, surrendered certain rights and powers and duties to the Union out of which the constitution was made. Of powers affecting health administration apparently they surrendered none, and need not, therefore, if they do not wish to, have any dealings with the Federal Health Department, save in so far as what they do or fail to do may lead to the involvement of other States.

### Fine Laboratory Work.

Quarantine and research are two main functions of this Federal Service. In Washington arrangements are made for research both in the field and in the laboratory. In the field, research is being carried out on a wide scale into particular diseases, ranging from cancer to the common cold. Apart from the Rockefeller Institute in New York, the laboratory at Washington pleased and stimulated the visitors as much as anything which they saw :—

It appeared to be so full of possibilities; it was so well organised; there was so much of fining down to the investigation by one individual or group of individuals of one particular matter. Here, in England, it has always seemed to me that no one ever gets a chance to go into anything; there is so much routine work to be done that the worker cannot get away from it to try experiments that may lead to something new.

### The Man with the Gun.

At Richmond, in the State of Virginia, Dr. Porter found much to interest him, and waxes particularly enthusiastic about the milk supply; he says that in the matter of clean milk production Richmond is years ahead of any town in Great Britain. But Virginia is largely rural, and was visited mainly for the examination of problems in rural hygiene. The great problems have been the filth diseases, typhoid fever, and "book-worm disease," and malaria. To get rid of the first two the chief necessity has been to abolish insanitary methods of disposing of filth and to improve the water supply. Before anything could be done it was essential to educate the people, who were extraordinarily ignorant and bigoted. This has proved no easy task :—

The people who live in the more remote parts are very far behind, and even when they can be got at—the roads generally being very bad—resent any attempt to teach them, quite commonly showing a tendency to use a gun to make clear their point of view. The life of a health officer, since the introduction of prohibition, in out-of-the-way places particularly, has been far from pleasant, more especially if he should find himself in the neighbourhood of a "moonshine" factory.

### A Dry Country.

In regard to prohibition generally in the Northern States, Dr. Porter prefers to express no opinion. He allows himself, however, one or two pertinent observations :—

Most of the drinking which I have seen openly or secretly on the street or in hotels or restaurants has not been convivial, but almost entirely with the object of getting drunk. Since coming to the Northern States I have seen more people completely and hopelessly inebriated, at all hours of the day, than I have seen for years at home.

### Milk Lessons from the States.

Not only at Richmond, but elsewhere, was much to be learned in regard to milk supply. "If, "says Dr. Porter, "I was undeceived in anything, it was with regard to milk, and without hesitation I say that the conditions in the case of milk are in general better in the United States than here, and probably than in any European country, and that the bulk of milk consumers in American cities of any size get a safer article." For example, in Boston practically all the milk is pasteurised and sold in bottles :—

The powers of the Commissioner of Health in regard to milk are very great, and the practice of sampling both for chemical and bacteriological examinations much more thorough than with us. The fat standard is higher than that in England, and from the records I studied at the laboratories in Boston it appears to be certain that the average milk on the market is richer in fat than ours. Also it contains infinitely fewer bacteria. One creamery near Boston that I visited greatly impressed me. The machinery for washing bottles and for bottling and sterilising the milk was of the type formerly used in breweries here and worked perfectly.

### Milk Education and a Public Spirit.

The authorities began with propaganda work, educating the people up to the need for milk and the necessity for a good, pure, clean supply. In this latter they have been very greatly assisted by the trade, though at first they were not quite so ready to assist, and, indeed, in many places offered rather strenuous opposition to the efforts of the authorities. The Boards of Health and Commissioners did not hesitate to use their very considerable powers. The railroad and other distributing agencies have cooperated, and generally there was found a public-spiritedness and a determination to ensure a pure and clean milk supply. One very definite claim made as the result of this is that there is an undoubted decrease in tuberculosis of the bones, joints, glands, &c.—the so-called "surgical tuberculosis"—among children.

### Maternity Work.

The great diminution in infantile diarrhœa in the States is attributed to the fact that so high a percentage of the milk sold is pasteurised. Maternity work is not, however, in general a subject on which we have much to learn from the States. In the South a very great part of the work is done by coloured midwives, who in the main are intensely ignorant and illiterate, there being no provision for the training of midwives, and a great unwillingness to provide for training. In Boston no one but a medical practitioner may report a birth, which is taken to mean that no one but a medically qualified person may attend a woman in labour. Despite all this it is believed that many women do practise midwifery and that many women have none but a woman to attend to them, though the reports of births, in accordance with the law, are made by medical practitioners.

### Food Protection.

In the Federal Laws the provision made for meat and food inspection is most full and complete, and in general the claim that has been made for the system, that "in its scope and magnitude it stands alone among the meat inspection systems of the world," is probably justified, and the statement that it is "a service in hygiene and sanitation of incalculable value to the nation," and that "its benefits extend to those foreign countries that receive meat from the United States," probably accurate:—

Why the Americans should be so outstandingly careful and we so dreadfully careless with food; they so determined to handle food with respect and to protect it from contamination, and we consumed with anxiety to ensure that no opportunity shall be lost for any and everything we eat to pick up all the dirt, filth and foulness it can, is not at all clear. So far as butchers' shops are concerned, except in poor alien quarters of New York and Boston, I never saw anything resembling an English butcher's shop anywhere in the States I visited. Nor did I see a fishmonger's stall on the pavement or a coster peddling fish or meat or, if I remember rightly, vegetables. Bread also I found to be more carefully protected. In certain places I visited, bread, cakes, &c., could only be exposed if enclosed in glass cases, and wrapping of bread was insisted upon.

### Ellis Island.

Dr. Porter's concluding notes on Ellis Island are of interest in view of the complaints that have been given so much publicity from time to time:—

All that we saw was, of course, on the medical side and seemed to be excellently arranged and so as to cause no unnecessary inconvenience. The work was rapidly done and the person who passed the examination was free to go in about three hours at the outside, provided the immigration authorities, of whose work we saw nothing, were satisfied. The object of the medical examination is to discover whether or not the persons are suffering from diseases that might spread if they were allowed to enter, or that will incapacitate the individual from earning his own living. It appears to me to be not at all unreasonable. The hospitals on Ellis Island are well kept, though some of the wards appeared somewhat crowded.

